# The SlHB8 acts as a negative regulator in tapetum development and pollen wall formation in Tomato

**DOI:** 10.1093/hr/uhac185

**Published:** 2022-08-25

**Authors:** Caiyu Wu, Yang Yang, Deding Su, Canye Yu, Zhiqiang Xian, Zanlin Pan, Hongling Guan, Guojian Hu, Da Chen, Zhengguo Li, Riyuan Chen, Yanwei Hao

**Affiliations:** Key Laboratory of Horticultural Crop Biology and Germplasm Innovation in South China, Ministry of Agriculture, College of Horticulture, South China Agricultural University, Guangzhou 510642, China; Key Laboratory of Horticultural Crop Biology and Germplasm Innovation in South China, Ministry of Agriculture, College of Horticulture, South China Agricultural University, Guangzhou 510642, China; Key Laboratory of Plant Hormones and Development Regulation of Chongqing, School of Life Sciences, Chongqing University, Chongqing, China; Center of Plant Functional Genomics, Institute of Advanced Interdisciplinary Studies, Chongqing University, Chongqing 400044, China; Key Laboratory of Horticultural Crop Biology and Germplasm Innovation in South China, Ministry of Agriculture, College of Horticulture, South China Agricultural University, Guangzhou 510642, China; Key Laboratory of Plant Hormones and Development Regulation of Chongqing, School of Life Sciences, Chongqing University, Chongqing, China; Center of Plant Functional Genomics, Institute of Advanced Interdisciplinary Studies, Chongqing University, Chongqing 400044, China; Key Laboratory of Horticultural Crop Biology and Germplasm Innovation in South China, Ministry of Agriculture, College of Horticulture, South China Agricultural University, Guangzhou 510642, China; Key Laboratory of Horticultural Crop Biology and Germplasm Innovation in South China, Ministry of Agriculture, College of Horticulture, South China Agricultural University, Guangzhou 510642, China; UMR990 INRA/INP-ENSAT, Université de Toulouse, Castanet-Tolosan, France; Key Laboratory of Horticultural Crop Biology and Germplasm Innovation in South China, Ministry of Agriculture, College of Horticulture, South China Agricultural University, Guangzhou 510642, China; Key Laboratory of Plant Hormones and Development Regulation of Chongqing, School of Life Sciences, Chongqing University, Chongqing, China; Key Laboratory of Horticultural Crop Biology and Germplasm Innovation in South China, Ministry of Agriculture, College of Horticulture, South China Agricultural University, Guangzhou 510642, China; Key Laboratory of Horticultural Crop Biology and Germplasm Innovation in South China, Ministry of Agriculture, College of Horticulture, South China Agricultural University, Guangzhou 510642, China

## Abstract

Pollen development is crucial for the fruit setting process of tomatoes, but the underlying regulatory mechanism remains to be elucidated. Here, we report the isolation of one HD-Zip III family transcription factor, *SlHB8*, whose expression levels decreased as pollen development progressed. *SlHB8* knockout using CRISPR/Cas9 increased pollen activity, subsequently inducing fruit setting, whereas overexpression displayed opposite phenotypes. Overexpression lines under control of the 35 s and p2A11 promoters revealed that SlHB8 reduced pollen activity by affecting early pollen development. Transmission electron microscopy and TUNEL analyses showed that SlHB8 accelerated tapetum degradation, leading to collapsed and infertile pollen without an intine and an abnormal exine. RNA-seq analysis of tomato anthers at the tetrad stage showed that SlHB8 positively regulates SPL/NZZ expression and the tapetum programmed cell death conserved genetic pathway DYT1–TDF1–AMS–MYB80 as well as other genes related to tapetum and pollen wall development. In addition, DNA affinity purification sequencing, electrophoretic mobility shift assay, yeast one-hybrid assay and dual-luciferase assay revealed SlHB8 directly activated the expression of genes related to pollen wall development. The study findings demonstrate that *SlHB8* is involved in tapetum development and degradation and plays an important role in anther development.

## Introduction

Pollen development consists of two phases: microsporogenesis and microgametogenesis [1, 2]. During microsporogenesis, archesporial cells convert into pollen mother cells [1, 2]. The mother cells transformed into a tetrad of haploid microspores via meiosis [1, 2]. The tapetum, a layer of secretory cells, undergoes programmed cell death (PCD), releasing the callose and other cell wall-degrading enzymes into the locule; this divides tetrad into individual microspores by digesting the cell walls which are composed of the polysaccharide callose [3]. Defects in the tapetum generation and degradation usually result in pollen abnormal development and decreased fertility [4, 5].

In *Arabidopsis* and *rice*, the molecular mechanisms as well as key genes regulating tapetum development have been well illuminated [3, 6–11]. During early tapetum development, SPOROCYTELESS/NOZZLE (SPL/NZZ), EXCESS MICROSPORO CYTES1/EXTRASPORO GENOUS CELLS (EMS1/EXS), and TAPETUM DETERMINANT1 (TPD1) play key roles in determining the tapetal generation [12–15]. At the late microsporogenesis stage, the tapetum undergoes a tapetal cell death process that is controlled by the conserved genetic pathway DYT1–TDF1–AMS–MYB80–MS1 [11]. The loss-of-function mutants *dyt1*, *tdf1*, *ams*, and *ms1* show delayed tapetal degradation, whereas the *myb80* mutantdisplays a precocious tapetum degeneration phenotype [8, 16–20]. The pollen wall increases microspore survival, helps pollen to resist environmental stresses and provides pollen–stigma recognition. The pollen wall consists of exine and intine layers [21]. The exine, which is made up of lipid-like materials is synthesized by the sporophytic tapetum. Defectives in tapetum development and degradation result in pollen wall malformations [21]. *TDF1*, *AMS*, *MYB80*, and *MS1* are also involved in pollen wall development, with different roles in exine and sexine formation and transcription regulation of tapetum-specific genes related to pollen wall development such as *MS188*, *TEK*, *ABDG26*, *exl5/6*, *CYP703A2*, *LAP5/6*, and so on [10, 16, 20, 22–24].

In tomato, few reports have been published on the genes involved in pollen development, and among them, even fewer have examined the regulators of the conserved PCD genetic pathway [17, 25–32]. *SlDYT1* (*MS10*) was the first isolated regulator in the tomato tapetum development; the *ms10* mutant fails to produce fertile pollen, and all PCD pathway members as well as genes related to sporopollenin synthesis are down-regulated [17]. Another *Arabidopsis* homolog gene *bHLH89/90* isolated was *MS32*, whose loss-of-function resulted in delayed tapetum degradation. In addition, the genes involved in PCD are all down-regulated in the *ms32* mutant [26]. Moreover, *SlPIF4* was reported to induce pollen activity under cold conditions by interacting with the conserved tapetum PCD genetic pathway [25]. Although the conserved PCD genetic pathway was predicted to be present in tomato, other regulators involved in this process remain to be clarified.

The class III homeodomain-leucine zipper (HD-Zip III) transcription factor family were reported to determine the ad/abaxial polarity of leaves, anthers, vascular organs, and developing embryos [13, 33–37]. Five HD-Zip III genes including *PHABULOSA* (*PHB*)/*ATHB14*, *REVOLUTA* (*REV*), *PHAVOLUTA* (*PHV*)/*ATHB9*, *INCURVATA4*/*CORONA*/*ATHB15*, and *ATHB8* were identified in the Arabidopsis genome [38]. These HD-Zip III genes show overlapping expression and exhibit redundant functions. Single loss-of-function of these genes gives non-obvious phenotypes. Simultaneous mutation of REV, PHV, and PHB affected meristem formation and seedlings structure [38, 39]. HD-Zip III mRNA can be degraded by the microRNA *miR165/6* [40]. Up-regulation of HD-Zip III genes via disruption of microRNA regulation sites result in strong developmental phenotypes [37, 41, 42]. Gain-of-function of PHB and PHV displayed leaves with damaged polarity and abnormal appearance [41]. MiR166-PHB-SPL/NZZ module regulates the stamen polarity through modifying the boundary thickness [13]. In cucumber, CsSPL formed a complex with CsPHB and CsWUS to orchestrate sex organ development in an unidentified regulatory pathway [12]. There are six HD-Zip III genes in tomato, including *Solyc11g069470* (*SlREV*), *Solyc08g066500* (*SlHB8*), *Solyc12g044410*, *Solyc03g120910* (*SlHB15A*), *Solyc02g024070*, and *Solyc02g069830* [43]. These genes are negatively regulated by *miR166*; overexpression of Sly-pre-miR166b down-regulates all six HD-Zip III genes, and the plant bears a fruit with another fruit developing inside or parthenocarpic fruit [44]. Overexpression of *SlREV* does not result in a discernable plant phenotype, but its overexpression via disruption of microRNA regulation leads to ectopic flower formation and fused fruit [44]. SlHB15A regulates parthenocarpic fruit set under cold conditions via *miR166*-mediated recessive dosage sensitivity [43]. Although the effect of SlREV and SlHB15A genes on fruit development has been clarified, its role in anthers need to be investigated.

Here, we isolated one HD-Zip III family transcription factor, SlHB8, whose loss-of-function increased pollen activity, inducing fruit setting. *SlHB8* overexpression showed the opposite phenotype. In order to clarify the function of SlHB8 in pollen development, cytobiology and molecular biology technologies were carried out on the anthers at different development stages. The results proved SlHB8 was a negative regulator during pollen development by mediating the tapetum development through conserved genetic pathway DYT1–TDF1–AMS–MYB80.

## Results

### *SlHB8* shows a pollination-dependent expression pattern

**Figure 1:**
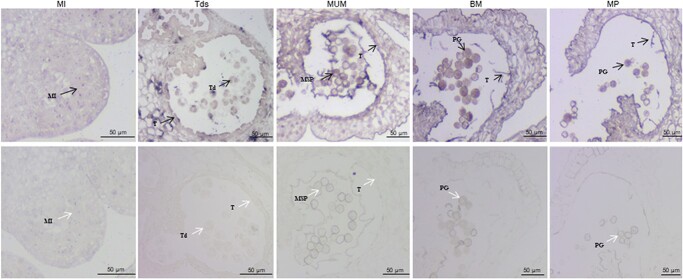
RNA in situ hybridization of *SlHB8* during anther development of wild-type tomato plant. Anthers at MI, Tds, MUM, BM and MP stages were cross-sectioned for hybridization with antisense (upper) and sense (lower) probes of *SlHB8*. Black and white arrows indicate positive and negative in situ hybridization signals for *SlHB8* transcripts respectively. BM: binucleate microspore stage; MI: microspore mother cell stage; MP: mature pollen stage; MSP: microspore pollen; MUM: middle uninucleate microspore stage; PG: pollen grain; T: tapetum; Td: tetrad; Tds: tetrad stage. Bars = 50 μm.

The SlHB8 transcription factor belongs to the HD-ZIP III transcription factor family, as it contains the four conserved domains: HD, bZip, START, and PAS [45]. *SlHB8* is expressed in all tissues, such as the root, stem, leaves, flowers, mature green fruits, breaker fruits, and red fruits, showing highest expression level in stem and lowest expression level in the red rip fruit (Fig. S1a, see online supplementary material). The highest expression level in the stem is in line with the enlarged stem diameter of *SlHB8* knocking out lines [46]. Here, *SlHB8* was detected in the petal, sepal, stamen, and carpel flower organs and showed the highest expression level in sepals, as evidenced by qPCR (Fig. S1b, see online supplementary material). During stamen development, *SlHB8* expression decreased (Fig. S1c, see online supplementary material). The *in situ* hybridization result showed that *SlHB8* transcripts were found in the microspores and tapetum cells from microspore mother cell stage to the mature pollen stage (Fig. 1), indicating its role in the pollen development. *SlHB8* is induced after treatment with auxin, gibberellic acid, and artificial pollination [45]. When the fruit set succeeded, *SlHB8* expression levels increased when compared to ovaries before pollination (Fig. S1d, see online supplementary material). These data suggest that *SlHB8* is important for stamen development and the fruit setting process.

### Knocking out *SlHB8* via CRISPR/Cas9 promotes fruit set rate and pollen activity in tomato

**Figure 2:**
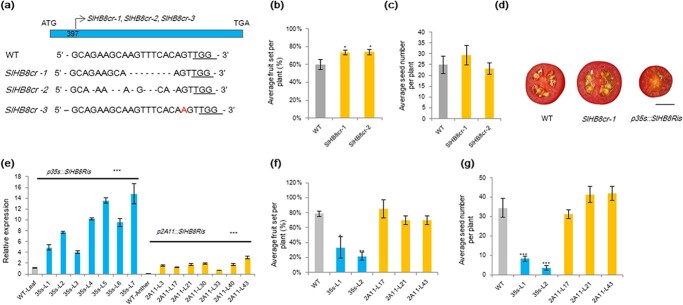
Phenotyping of *SlHB8* gene overexpression and knockout plants. **a** Three *SlHB8* gene knockout lines were established using CRISPR/Cas9. Fruit set rate (**b**) and the number of seeds per fruit (**c**) of *SlHB8* knockout plants. **d** Photos of cross-section of red ripe fruits of wild-type, *SlHB8* gene knockout and *SlHB8* overexpression lines. Scale bar = 1 cm. **e** Expression levels of *SlHB8* under control of the 35 s and 2A11 promoters in the *SlHB8* overexpression lines. *Ubi* was used as a reference gene. Fruit set rate (**f**) and the number of seeds per fruit (**g**) of *SlHB8* gene overexpression plants. The error bars denote SE; ^*^P < 0.5, ^**^P < 0.01, ^***^P < 0.001 (Student’s *t*-test; compared with the WT). 35 s-L1, promoter 35 s-driven *SlHB8* overexpression line 1; *2A11-L17*, promoter 2A11-driven *SlHB8* overexpression line 17.

**Figure 3:**
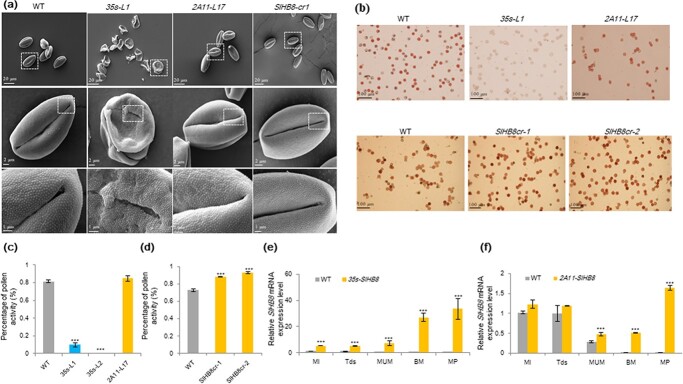
Pollen viability and pollen morphology of *SlHB8* gene knockout and overexpression lines. **a** Scanning electron micrographs of pollen grains from wild-type, *SlHB8* gene knockout and *SlHB8* overexpression lines under control of the 35 s and 2A11 promoters. BM: binucleate microspore stage; MI: microspore mother cell stage; MP: mature pollen stage; MUM: middle uninucleate microspore; Tds: tetrad stage. From the top to the bottom, the scale bar indicated 20 μm, 2 μm, 1 μm, respectively. **b** Pollen viability after TTC staining. Scale bar = 100 μm. **c, d** Percentage of pollen viability in wild-type and *SlHB8* transgenic lines. **e, f** Expression levels of *SlHB8* in the transgenic tomato lines *p35s::SlHB8Ris* and *p2A11::SlHB8Ris* during anther development. *Ubi* was used as reference gene. Expression level in MI stage flower bud was used as control. The error bars denote SE; ^***^P < 0.001 (Student’s *t*-test; compared with the WT).

To determine its role in anther development and the fruit setting process, we knocked out *SlHB8* using CRISPR/Cas9; *SlHB8*-specific primers and sequencing were used to verify the knockout effect. Three mutant types were obtained, including two with an 8 bp deletion in the CDS and one with a 1 bp insertion (Fig. 2a). Phenotyping was performed for the two deletion lines. Compared with the WT, the fruit set rates were higher in the *SlHB8* knockout lines (Fig. 2b), but the fruit size, weight, and seed number (Fig. 2c and dFig. S2a and b, see online supplementary material) did not change. Pollen activity, pollen tube length, and the pollen tube germination rate were higher in the *SlHB8* knockout lines than that in the WT (Fig. 3a,b–d, Fig. S2c–e, see online supplementary material), and the anther width was thinner (Fig. S2f, see online supplementary material).

### Overexpression of *SlHB8* results in pollen abortion and seedless fruits

An *miRNA166* target site was found in the *SlHB8* gene, and thus individual *SlHB8* overexpression lines under control of the 35S and fruit-specific 2A11 promoters were generated by mutating the *miRNA166* target site [47]; transgenic plants were verified by qPCR, with seven *p35S::SlHB8Ris* and seven *p2A11::SlHB8Ris* lines showing overexpressed *SlHB8* levels (Fig. 2e). Most *p35s::SlHB8Ris* overexpression lines produced seedless fruits (Fig. 2d); therefore, only two lines (line 1 and 2) were collected from a few seeds [47]. Compared with the WT, the *p35::SlHB8Ris* overexpression transgenic plants bore fewer fruits (Fig. 2f) and the fruit size, weight, and seed number (Fig. 2g, Fig. S3a and b, see online supplementary material) were significantly reduced. All *p2A11::SlHB8Ris* overexpression lines displayed phenotypes – such as fruit set, seed number, fruit size, and fruit weight – similar to those of the WT tomato plants (Fig. 2f and g, Fig. S3a and b, see online supplementary material).

**Figure 4:**
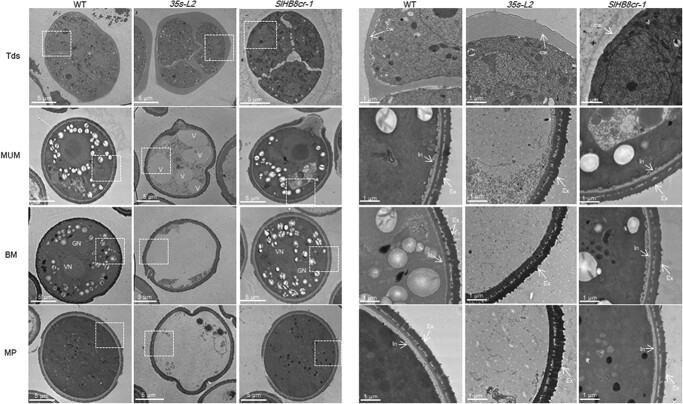
Transmission electron microscopy of pollen development in the wild-type, *SlHB8* gene knockout and *p35s::SlHB8Ris* lines. BM: binucleate microspore stage; cw: callose wall; Ex: exine; GN: generative nuclei; In: intine; MP: mature pollen stage; MUM: middle uninucleate microspore stage; Tds: tetrad stage; V: vacuole; VN: vegetative nuclei. The scale bar indicated 5 μm (left: whole organ) and 1 μm (right: magnified location), respectively.

Because pollen viability and ovule development affects the fruit set rate and seed number, which were reduced in the L1 and L2 lines, reciprocal cross experiments between *p35s::SlHB8Ris* and the WT as well as *p2A11::SlHB8Ris* and the WT were performed. When the WT was used as the female parent and *p35s::SlHB8Ris* as the male parent, the fruit set rate reached 2.7%; however, the fruit set rate decreased to 93.5% when this was switched (Table 1). In contrast to *p35s::SlHB8Ris*, the fruit set rate remained similar regardless of the pollen donor when WT and *p2A11::SlHB8Ris* were crossed (Table 1). These results indicate that the pollen varieties were mainly responsible for the lower fruit set rate and reduced seed number. We thus assessed the viability of mature pollen grains at the anthesis stage using the TTC method. Via microscopy, pollen grains of *p35s::SlHB8Ris* were found defective, whereas those of *p2A11::SlHB8Ris* and *SlHB8-cr* were similar to those of the WT (Fig. 3a and b). In addition to pollen viability, pollen shape, pollen tube length, and pollen tube germination capacity showed observable differences between *p35s::SlHB8Ris* and the WT (Fig. 3a–c, Fig. S3c–e, see online supplementary material), and the *p35s::SlHB8Ris* anther width was thinner (Fig. S3f, see online supplementary material). By contrast, pollen viability, pollen tube length, pollen tube germination capacity, and morphology of the *p2A11::SlHB8Ris* lines were similar to those of the WT (Fig. 3a–c, Fig. S3c–e, see online supplementary material). SEM was then employed to observe the whole structure of mature pollens at the anthesis stage. Unlike the round and regularly shaped WT pollen grains, the *p35s::SlHB8Ris* transgenic pollen grains were irregular, shrunken, and collapsed. The pollen grain surface was also different from that of the WT (Fig. 3a). No significant differences were observed between *p2A11::SlHB8Ris*, *SlHB8cr*, and the WT (Fig. 3a). Next, transmission electron microscopy (TEM) was used to investigate the ultrastructural changes of the aborted *p35s::SlHB8Ris* pollen grains; the exine layer of irregular pollen grains was wider in *p35s::SlHB8Ris* than in the WT, and the intine layer between the plasma membrane and exine was absent in the *p35s::SlHB8Ris* lines (Fig. 4).

**Table 1:** Cross-fertilization assay.

	**SlHB8-ox♂ x WT♀**	**WT♂ x SlHB8-ox♀**	**2A11-SlHB8♂ x WT♀**	**WT♂ x 2A11-SlHB8♀**
Fruit set	2.70%	93.50%	60.60%	50%
Hybrid number	37	31	33	30
Setted fruits number	1	29	20	15

Emasculated wild type flowers were fertilized with SlHB8-ox pollen and the number of setting fruit [lr1] was assessed at the ripe stage. Conversely, tomato pollen from wild type flowers was used to fertilize emasculated SlHB8-ox flowers. The same assay was also carried out on the 2A11 lines.

Because of the different effects of *SlHB8* on fruit morphology in the *p35s::SlHB8Ris* and *p2A11::SlHB8Ris* overexpression lines, we compared *SlHB8* expression levels during anther development. The *SlHB8* gene was overexpressed throughout anther development (from MI to MP) in *p35s::SlHB8Ris* (Fig. 3e), but was induced starting from the MUM stage in *p2A11::SlHB8Ris* (Fig. 3f). This differential expression pattern may account for the development of seedless fruit in *p35s::SlHB8Ris* transgenic plants.

### Overexpression of *SlHB8* disrupts pollen development due to early tapetal PCD

**Figure 5:**
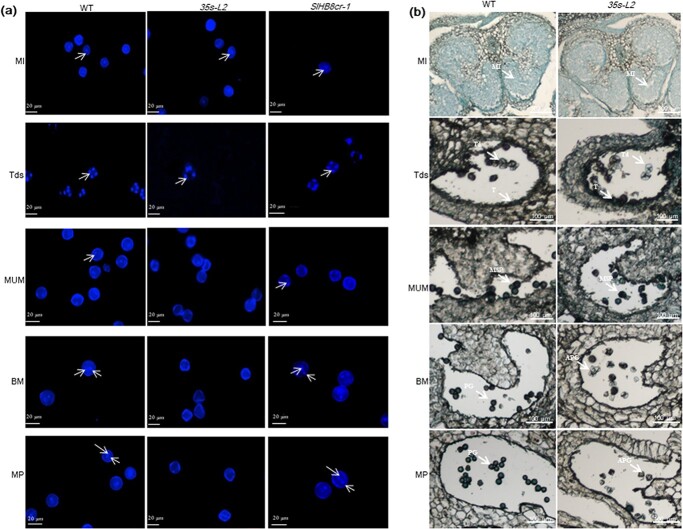
Histocytological observation of pollen development in the wild-type, *SlHB8* gene knockout and *SlHB8* overexpression lines under control of the 35 s promoter. DAPI staining (**a**) and semi-thin section comparison (**b**) of anther and pollen development between the wild-type, *SlHB8* gene knockout and *35 s-L2* lines. APG: abnormal pollen grain; BM: binucleate microspore stage; MI: microspore mother cell stage; MP: mature pollen stage; MSP: microspore; MUM: middle uninucleate microspore stage; PG: pollen grain; T: tapetum; Td: tetrad; Tds: tetrad stage. The white arrows indicate the nuclei (**a**). The scale bar indicated 20 μm (**a**) and 100 μm (**b**).

**Figure 6:**
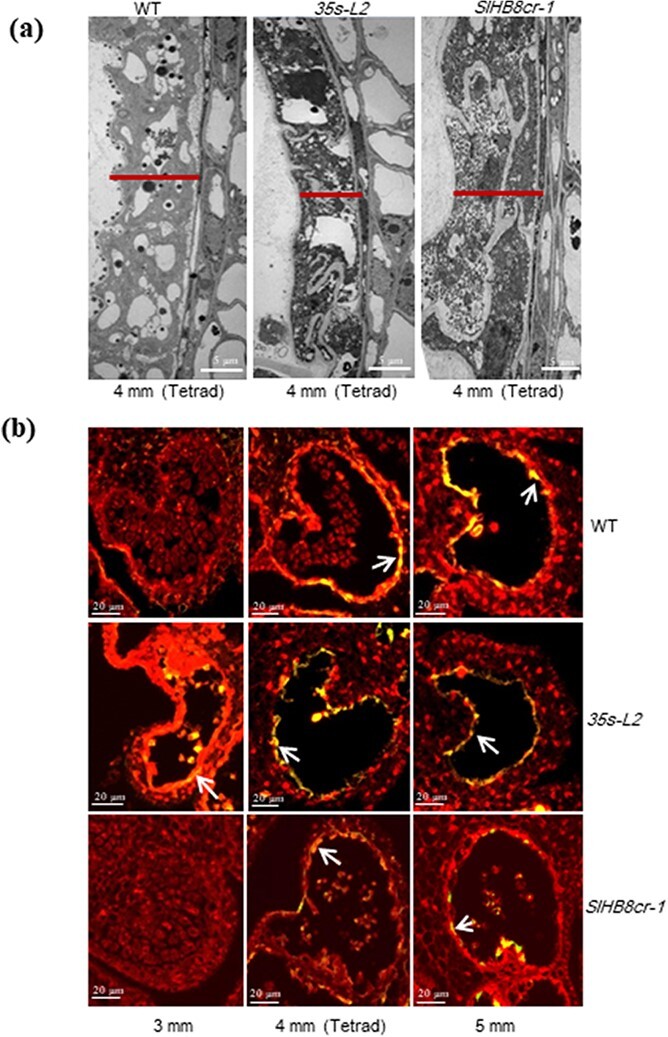
Morphology of tapetum development and programmed cell death. **a** Transmission electron micrographs of anthers at the tetrad stage of wild-type, *SlHB8* gene knockout and *SlHB8* overexpression plants under control of the 35 s promoter. Scale bars = 5 μm. **b** Fluorescence microscopy of DNA fragmentation detected by TUNEL assays of anthers from wild-type, *SlHB8* gene knockout and *35 s-L2* line plants at different stages. Scale bars = 20 μm.

To identify the key stage at which SlHB8 regulates pollen development, we defined the pollen development stages as MI, Tds, MUM, BM, and MP (anthesis) using DAPI staining (Fig. 5). At the MI and Tds, the nuclei and tetrad formed normally both in the WT, *SlHB8cr* and *p35s::SlHB8Ris* transgenic lines. Starting from the MUM stage to the MP stage, the nucleus disappeared in the *p35s::SlHB8Ris* lines and the pollen shape became irregular and collapsed, which is obviously different from that of WT and *SlHB8cr* anthers with two nuclei and round, flush pollen grains (Fig. 5a). We further performed a set of cytological experiments to characterize the spatial and temporal cytological defects in *p35s::SlHB8Ris* anthers. In agreement with the above observations, histological anther sections showed that at the MI stage, the cell layer differentiation appeared similar to that of WT anthers, and a tetrad formed in both the WT, *SlHB8cr* and *p35s::SlHB8Ris* anthers during the Tds; there were no observable defects in the anthers during these two stages (Fig. 5b, Fig. S4, see online supplementary material). At the MUM stage, abnormal pollen grains with an irregular shape and vacuolation appeared in the *p35s::SlHB8Ris* anthers, and most of the pollen grains were aborted in the *p35s::SlHB8Ris* lines (Fig. 5b). Via TEM, we observed a large nucleus in the WT and *SlHB8cr*, which was not present in the *p35s::SlHB8Ris* lines (Fig. 4). Instead, there was an increased number of large vacuoles, and the intine of the pollen wall was absent during the MUM stage (Fig. 4). During the WT and *SlHB8cr* BM stage, the microspore contained a full cytoplasm with normal vegetative and generative nuclei (Fig. 4). However, the majority of the cytoplasm and nuclei in pollen grains of the *p35s::SlHB8Ris* lines were completely degraded, and only trace cytoplasmic inclusions were observed (Fig. 4). At the Tds, the *p35s::SlHB8Ris* anther tapetum was thinner than that of the WT and *SlHB8cr-1*, and tapetal cells were infertile and shrunken (Fig. 6a).

Tapetum PCD is one of the key factors affecting tapetal cell degradation and pollen development. To assess whether the infertile tapetal cells are associated with early PCD, we performed a TUNEL assay, which provides strong fluorescent signals when cells undergo massive DNA fragmentation. In the WT and *SlHB8cr-1*, a positive signal appeared at the 4 mm anther stage (Tds), which was observed earlier in *p35s::SlHB8Ris* at the 3 mm anther stage (Fig. 6b). The results indicate that *SlHB8* overexpression accelerates tapetal cell degradation, leading to pollen abortion.

### Primary metabolite determination in *p35s::SlHB8Ris* anthers at the mature pollen stage

Given that anther development is associated with metabolite levels, we determined primary metabolite content in the WT and *p35s::SlHB8Ris* anthers. Primary metabolites measured included amino acids and derivatives; nucleotides and derivatives; carbohydrates; indole derivatives; organic acids and derivatives; and lipids. All nucleotides and derivatives were down-regulated, and with the exception of O-rhamnoside, levels of the other three carbohydrates measured were reduced. All lipids detected were significantly up-regulated, as well as half of the organic acids and derivatives, with the other half down-regulated. All indole derivatives were down-regulated in the *p35s::SlHB8Ris* anthers (Fig. S5; Table S1, see online supplementary material).

### Transcriptome analysis of DEGs after *SlHB8* overexpression

Given that SlHB8 belongs to the HD-Zip III transcription factor family and that its up-regulation resulted in pollen abortion and anther development defects, SlHB8 was predicted to regulate pollen development by mediating the transcription of target genes. To identify such genes, comparative transcriptome analyses via RNA-seq were performed using tetrad stage flower bud. Each sample contained three biological replicates, and totally six transcriptome libraries were generated. In our study, 97.17%–97.53% of short clean reads identified from RNA-seq data were mapped to the tomato genome (*Solanum lycopersicum* ITAG4.0). Among the six libraries, 23 723 to 24 399 genes were annotated and 659 novel genes were identified (Table S2, see online supplementary material). The correlation among the three biological replicates was qualified by Pearson’s correlation coefficient (*R^2^*), with *R^2^* > 0.8 as the significance cutoff. The *R^2^* value of the three biological replicates was >0.99, indicating a high correlation (Fig. S6, see online supplementary material).

**Figure 7:**
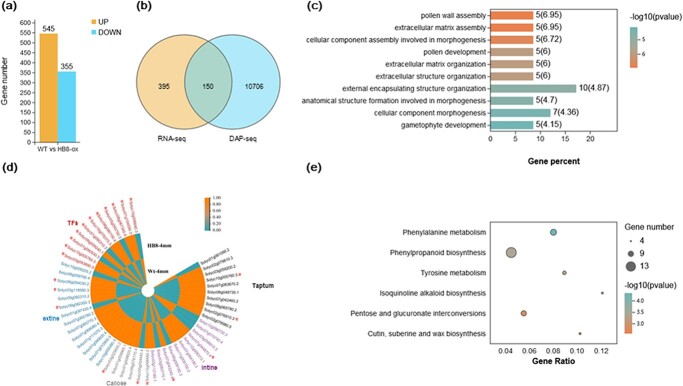
Identification of differentially expressed genes (DEGs) and SlHB8-targeted genes in wild-type and *p35s::SlHB8Ris* anthers by RNA-seq and DAP-seq. **a** DEGs between wild-type and *p35s::SlHB8Ris* anthers at the tetrad stage. **b** Overlap of DEGs identified by RNA-seq and SlHB8-target genes identified by DAP-seq. Gene ontology (GO) (**c**) and Kyoto Encyclopedia of Genes and Genomes (KEGG) (**e**) analysis of up-regulated DEGs in wild-type and *p35s::SlHB8Ris* anthers at the tetrad stage. **d** Heatmaps of DEGs involved in pollen development, including DEGs related to sporopollenin biosynthesis and transport, tapetum development, pollen intine development, callose metabolites, and transcription factors. ^*^ indicates up-regulated DEGs targeted by SlHB8.

To identify DEGs in tetrad stage anthers between the WT and *p35s::SlHB8Ris* lines, pairwise comparisons were performed with |log2 (fold change)| > 1 and FDR < 0.05 as cutoff thresholds to filter the significant DEGs. These comparisons allowed the identification of 900 DEGs, including 355 down-regulated and 545 up-regulated genes (Fig. 7a; Table S3, see online supplementary material). Among these 900 DEGs, genes involved in the regulation of microspore protein biosynthesis, tapetum development, callose metabolism, pollen inner wall formation, pollen outer wall formation, and hormone signal transduction pathways related to pollen development were up-regulated (Fig. 7d).

qRT-PCR was then performed using seven randomly selected genes to confirm the accuracy of RNA-seq. All seven genes exhibited similar expression pattern and high Pearson’s correlation coefficient [RNA-seq and qRT-PCR: 0.9715 (*P* < 0.0001)], indicating that the transcriptome data were highly reliable (Fig. S7, see online supplementary material).

As SlHB8 was predicted to be an activator, the up-regulated DEGs may represent a direct response of *SlHB8* overexpression. To further understand the putative functions of these 545 up-regulated DEGs, gene ontology (GO) assignment and Kyoto Encyclopedia of Genes and Genomes (KEGG) enrichment analysis were carried out. Using a significant cutoff value of q ≤ 0.05, the data revealed that the 545 DEGs were only significantly enriched in the biological process term related to pollen wall assembly, pollen development, and gametophyte development (Fig. 7c; Table S4, see online supplementary material), with six KEGG pathways significantly enriched (Fig. 7e; Table S4, see online supplementary material): ‘phenylalanie metabolism’, ‘tyrosine metabolism’, ‘isoquinoline alkaloid biosynthesis’, ‘phenylpropanoid biosynthesis’, ‘pentose and glucuronate interconversions’, and ‘cutin, suberin and wax biosynthesis’. The last three pathways have been proved to be related to pollen development (Fig. 7e).

### Identification of SlHB8-targeted DEGs by DAP-seq

**Figure 8:**
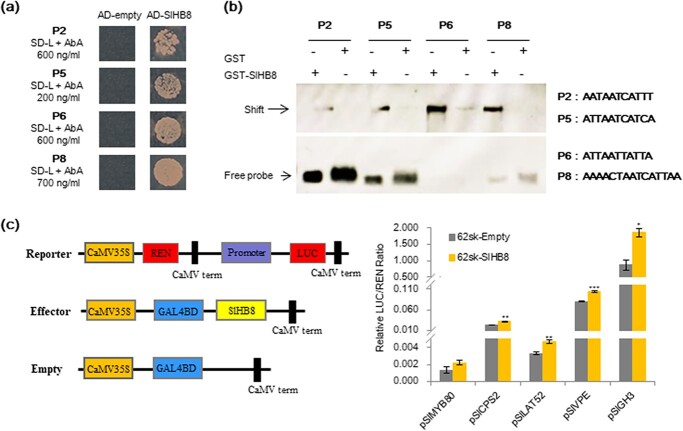
Validation of SlHB8 downstream target genes via yeast one-hybrid, EMSA and dual-luciferase assays. **a** Validation of SlHB8 binding with the four selected DAP-seq fragments using yeast one-hybrid assay. AD-empty indicates the control yeast strain transformed with the empty pGADT7 vector without SlHB8. **b** EMSA validation of the binding motifs in four selected DAP-seq fragments. The corresponding motif are listed beside the band. The detected bands are indicated with black arrows. **c** Validation of SlHB8 activation on five selected promoters using a dual-luciferase assay. The empty effector was used as control (set as 1). The ratio is presented as the mean ± SE (*n* = 3).

DEGs identified by RNA-seq were directly or indirectly regulated by SlHB8. To identify DEGs directly regulated by SlHB8, we used DAP-seq to identify the SlHB8-binding sites *in vitro*. In total, 71 504 200 bp peaks were identified in the tomato genome, including 12 627 (17.66%) peaks that were presented within 2.0 kb upstream of the annotated ORFs, 4583 (6.41%) located within 300 bp downstream of putative ORFs, and the remaining 54 293 (75.93%) distributed in introns, exons, or intergenic regions in the genome (Fig. S8; Table S5, see onlinesupplementary material). To confirm the DAP-seq results, we selected four binding elements for Y1H and EMSA assays. SlHB8 interacted with all tested elements (Fig. 8a and b). Furthermore, we performed a dual-luciferase assay using five genes (*SlVPE*, *SlCPS2*, *SlLAT52*, *SlGH3.1*, and *SlMYB80*) whose promoters contained the target motifs of SlHB8 and found that SlHB8 activated their expression except *SlMYB80* which showed a slight up-regulation without significance (Fig. 8c). After analyzsing the genes with promoters containing the DAP-seq fragments and the SlHB8-regulated DEGs, we revealed 150 overlapping genes (Fig. 7b; Table S6, see online supplementary material). Among these genes, there were genes involved in sporopollenin biosynthesis and transport, tapetum development, hormone metabolism and signaling pathways, pollen intine development, callose metabolism and genes belong to transcription factors, indicating that SlHB8 regulates pollen development by mediating these pathways (Fig. 7d; Table S6, see online supplementary material).

**Figure 9:**
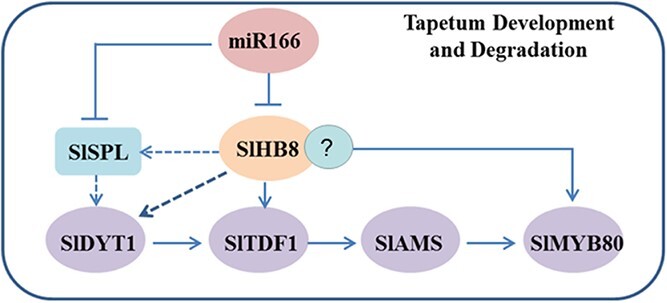
Schematic model of SlHB8-regulated genes in tapetum development and degradation.

## Discussion

### SlHB8 regulates pollen development by disturbing tapetum PCD

The tapetum and its degradation triggered by PCD which produced enzymes, nutrients, and precursors play an important role in microspore and pollen wall development [3]. Numerous reports have shown that incorrect timing of tapetal PCD (premature or delayed degradation) and disintegration induces male sterility and pollen wall defects [3, 6–9, 16, 29]. In contrast to previous results and our findings in the WT, tapetum PCD in the *SlHB8* overexpression lines occurred early in tomato anthers, before the tetrad stage, while tapetum PCD in the *SlHB8* gene knockout lines appeared at the tetrad stage (Fig. 6b).

Over the past decade, the functionally conserved genetic pathway DYT1–TDF1–AMS–MYB80 in tapetum development and degradation has been identified in *Arabidopsis*, rice, tomato, and other crops [3, 7, 11, 17, 26]. Loss-of-function of these regulators results in aborted pollen and induces male sterility [6, 8–10, 16, 17, 26]. DYT1 is a critical transcription factor for early tapetum development and function upstream of *TDF1*, *AMS*, *MYB80*, *TEK*, and *MS1* [16, 17]. *TDF1* is the direct target gene of DYT1, and mutations in *TDF1* severely impair tapetal development and callose dissolution. Tapetal cells in *tdf1* fail to transit to the secretory type as a result of the low expression levels of *MYB80* [9, 16]. AMS and MYB80 act as master regulators of pollen wall development due to its regulation on the genes related to callose degeneration (*QRT3* and *A6*), genes related to sporopollenin biosynthesis and metabolism (*CYP86C3*, *ACOS5*, and *SHT*), genes related to lipid transport (*LACS6* and *WBC27*), and pollen coat formation (*EXL4*-*EXL6*) [9, 16, 23]. Interestingly, our RNA-seq data showed that the core transcription factors that regulate tapetal PCD (*SlDYT1*, *SlTDF1*, and *SlMYB80*) and their multiple downstream targets (e.g. *SlA6*, *CYP703A2*, *CYP704B1*, *SlPKSA*, and *SlPKSB*) were up-regulated in the *SlHB8* overexpression lines (Fig. 7d); *SlAMS* was also significantly up-regulated (fold <2) (Table S7, see online supplementary material). Moreover, DAP-seq, Y1H, and dual-luciferase assays confirmed that some genes were directly targeted by SlHB8, such as *SlMYB80* and *SlA6* (Fig. 8; Table S6, see online supplementary material), indicating that SlHB8 induces tapetum PCD by regulating these key tapetal pathway genes. Moreover, in the *SlHB8* overexpression lines, TUNEL-positive signals were observed in the microspores before the tetrad stage (Fig. 6b), suggesting early callose generation and early secretory tapetal cell transition, which may induce early tapetum degradation. The overexpression of *SlMYB80* and *SlA6* also supports the hypothesis that early decretory tapetal cell transition leads to early tapetum PCD. While loss-of-function of SlHB8 has no effect on the tapetum PCD signal and expression of tapetal PCD regulators (Fig. 6b, Fig. S9c, see online supplementary material), indicating another gene may compensate the function of *SlHB8* or another partner work together with SlHB8 in regulating the tapetum PCD.

### SlHB8 may function upstream of *SlSPL* to regulate early pollen development

SPL has been reported to function in the process of sex organ development [12–14, 48, 49]. In *Arabidopsis*, SPL/NZZ controls early microsporocyte differentiation. Loss-of-function of SPL resulted in inhibited microsporocyte formation and deformed tapetum [13, 14]. In cucumber, CsSPL formed a complex with HD-Zip III and CsWUS to regulate anther and ovule development [12]. In tomato, the SlSPL loss-of-function mutant *hydra* showed sterility phenotypes both on male and female organs [49]. In developing anthers of *Arabidopsis*, *miR165/6* acts as a regulator balancing the expression of *PHB* and *SPL/NZZ* to determine the polarity of the anthers [13]. In addition to *PHB*, the adaxial identity genes include the other HD-Zip III family genes (*REV*, *PHB*, *PHV*, *CNA*, and *ATHB8*), which are also repressed by *microRNA165/6* [50]. Overexpression of *SlHB8* with a mutated *miR165* target site resulted in aborted pollen grains (Fig. 3a–c) and up-regulation of *SlSPL* (Table S3, see online supplementary material), indicating its upstream function during tomato anther development. According to the DAP-seq data, a non-SlHB8 binding site was found in the *SlSPL* promoter, indicating indirect regulation by SlHB8, a regulatory loop with *miR166*, or a loss of gene expression. *DYT1*, which is positively regulated by SPL and EMS1, functions downstream of SPL and partially rescues the *spl* phenotype [51]. DYT1 is sufficient to activate the downstream genes, such as *TDF1*, *AMS*, *MYB80*, *TEK*, and *MS1*, in tapetum development [16]. In the *p35s::SlHB8Ris* lines, the expression of *DYT1*, *TDF1*, *MYB80*, and *TEK* was induced (Fig. 7d). Furthermore, a SlHB8 binding site on the promoter of *SlMYB80* was identified, but its activation by SlHB8 was not strong (Fig. 8), indicating that SlHB8 needs a partner or that the elevated expression levels of *SPL*, *DYT1*, and *TDF1* contribute to the up-regulation of *SlMYB80* in the *p35s::SlHB8Ris* lines. Overall, the results indicate that SlHB8 may function upstream of *SlSPL* and regulates early pollen development.

### SlHB8 affects exine and intine development

The genetic pathway (DYT1–TDF1–AMS–MS188–MS1) for tapetum development is reported to be closely connected to exine formation in *Arabidopsis*. The key genes involved in sporopollenin formation, such as *CYP703A2*, *CYP704B1*, *PKSB*, and *PKSA*, are positively regulated by DYT1, TDF1, AMS, and MYB80 [23, 52]. Moreover, AMS regulates nexine and sexine layer formation by directly modifying the expression of *TEK* and *MS188*. The absence of *TEK* function results in pollen grains without the intine and exine layers [22]. In the present study, the exine and intine were affected in the *p35s::SlHB8Ris* lines, with an extine thicker than that of the WT and an absent intine (Fig. 4). Primary metabolite levels and the main components of sporopollenin were also altered in the *SlHB8* overexpression lines (Fig. S5, see online supplementary material). *SlDYT1*, *SlTDF1*, *SlMYB80*, *SlTEK*, *SlCYP703A*, *SlPSKB*, and *SlPSKA* were up-regualted when *SlHB8* was overexpressed (Fig. 7d), which is consistent with the abnormal pollen wall development of the *p35s::SlHB8Ris* lines. We did not find a SlHB8 binding site in the promoters of *SlCYP704B1*, *SlCYP703A*, *SlPSKB*, and *SlPSKA*; however, such a binding site was present in the *SlMYB80* and *SlTEK* promoters (Table S5, see online supplementary material), indicating indirect up-regulation resulting from the elevated expression levels of *SlMYB80* and *SlTEK*, which target *CYP703A2*, *CYP704B1*, *PSKB*, and *PSKA* in *Arabidopsis*.

Compared with exine development, knowledge of intine formation is lacking. Intine is secreted by the microspores and is associated with pectin, cellulose, and callose metabolism [21]. Inhibition of intine synthesis during the early stages of male gametogenesis may arrest pollen development, leading to collapsed, aborted pollen [53–55]. As the pollen tube consists of an intine layer, defects of the intine structure are accompanied by abnormal pollen tube germination [54, 56–58]. In the *p35s::SlHB8Ris* lines, an intine layer did not form in the shrunken, irregular, and infertile pollen grains and the germination rate was reduced (Fig. 4; Fig. 3a, Fig. S3d, see online supplementary material). UDP-sugar pyrophyllase (USP) is involved in pectin synthesis; loss-of-function of AtUSP blocks the synthesis of matrix polysaccharides, which are required for intine synthesis, resulting in pollen without an intine layer [57]. The homologous gene of *USP* in tomato was found to be a direct target of SlHB8 (Table S5, see online supplementary material), but its expression level was not altered in the *SlHB8* overexpression line (Table S7, see online supplementary material), indicating a complex regulation of this gene. *PME* genes encode enzyme called pectin methylesterases which function in the de-esterification of pectin. *PME* genes belong to a multigene family, and some of them display a pollen-specific expression pattern. BcPME37c and BcMF23a mutations cause abnormal thickening of the pollen intine of *Bactris campestris*, which affects pollen germination and growth [54, 56]. Loss-of-function of PME48 leads to late pollen germination and lower germination rate [59]. In our study, four out of eight *PME* genes showed increased expression levels in the *p35s::SlHB8Ris* line (Fig. 7d), among which *SlPME8* was the target gene of SlHB8 (Table S5). Polygalacturonase (PG) – whose gene family is expressed in the pollen and/or anthers – functions in the pectin degradation and cell wall disintegration. BcMF2, BcMF6, and BcMF9 are associated with intine development. Inhibition of BcMF2 or BcMF9 results in reduced PG activity and disturbed pectin metabolism in the process of intine formation [53]. In our study, two out of three PG genes showed increased expression levels in the *p35s::SlHB8Ris* line (Fig. 7d), among which *SlPG1* was the target gene of SlHB8 (Table S5, see online supplementary material). PLLs encode Pectate lyases (or pectate transeliminases; PLs) function in the process of cell wall disintegration and are necessary for intine loosening. Down-regulation of *BcPLL9* led to abnormal intine formation and delayed pollen tube growth in *B. campestris* ssp. *chinensis* [60]. The reduced expression level of *BcPLL20* resulted in abnormal disproportionated of intine distribution [61]. In our study, two genes encoding the PLs (*LAT56* and *AT59*) were down-regulated in the *p35s::SlHB8Ris* line (Fig. 7d), suggesting the potential regulation by SlHB8. Finally, the fasciclin-like arabinogalactan protein affects microspore development and intine formation through cellulose deposition. Down-regulation of *FLA3* in plants reduces male fertility and produces collapsed pollens grains without an intine [55]. Antisense RNA transgenic lines with reduced *BcMF18* levels show abnormal pollen grains lacking an intine, cytoplasm, and nuclei as well as abnormal cellulose distribution [62]. In our study, two *FLA* genes showed increased expression levels in the *p35s::SlHB8Ris line* (Fig. 7d), but none contained a SlHB8 binding site (Table S5, see online supplementary material), indicating indirect regulation. Until now, most studies on intine formation have focused on the enzymes involved in pectin, cellulose, and callose metabolism. SlHB8 is thus the first transcription factor demonstrated to exhibit direct regulation of the genes involved in intine formation.

In summary, *SlHB8* exhibited space–time characteristic expression pattern from microsporocyte differentiation to the microspore generation (Fig. 1), phenotypes of the *SlHB8* knockout and overexpression lines along with the RNA-seq and DAP-seq data support the notion that SlHB8 together with the conserved genetic pathway SPL–DYT1–TDF1–AMS–MS80 are instrumental in the regulation of tapetum development and degradation. Loss-of-function of SlHB8 induces pollen activity and promotes fruit setting (Figs 2b and 3b,d), but the pollen morphology and tapetum degradation were similar to that of wild-type plant (Figs 3a, 4 and 5). Moreover, the expression levels of tapetum PCD regulators were not affected in the *SlHB8cr* plant (Fig. S9c, see online supplementary material) indicating another partner may compensate the function of SlHB8 or work together with SlHB8 in regulating the PCD process. By contrast, *SlHB8* overexpression induces the expression of these conserved pathway genes, leading to premature tapetum degradation and resulting in pollen abortion. Based on these findings, a putative regulatory mechanism was proposed (Fig. 9), where overexpression of *SlHB8* resistant to *miR166*-induced *SPL* expression directly or indirectly activates *DYT1*, *TDF1*, and *MYB80* expression, thereby accelerating tapetum development and degradation. Therefore, SlHB8 emerges as a factor regulating pollen development. These findings expand our understanding of the molecular factors involved in tapetum development and degradation and provide potential genes for breeding strategies aimed at controlling this important trait.

## Materials and methods

### Plant material and growth conditions

*SlHB8* knockout mutants were generated using CRISPR/Cas9. One single guide (sg) RNA (GCAGAAGCAAGTTTCACAGT) complementary to the coding sequence (CDS) of Solyc08g066500 was constructed into the pAGM4723 vector and transformed into *Agrobacterium tumefasciens*, which was used for tomato genetic transformation. Plants bearing two kinds of 8 bp deletions in the CDS and a 1 bp insertion in the CDS were obtained. The two 8 bp deletion lines were used for flower and fruit development studies. The overexpression lines *p35s::SlHB8Ris* and *p2A11::SlHB8Ris* were generated separately. The full-length CDS of *SlHB8Ris* was cloned into the overexpression vectors pMDC32 and 2A11, which were under control of the 35 s and 2A11 promoters, respectively, that show specific expression during the mature stages of anther and early fruit development [63]. The transgenic plants were selected on MS medium with antibiotic selection of the construction vector. Positive overexpression lines were identified by checking the expression levels of *SlHB8*. Experiments were conducted in an artificial climate room (25 ± 1°C) with a light:dark cycle of 16 h:8 h at the South China Agricultural University. The planting medium was a mixture of 2:1 imported peat soil and vermiculite, and plants were potted in a 10 × 10 cm planting container. Tap water with Huabao nutrient particles was used for daily watering to provide nutrition.

### RNA in situ hybridization

The anthers at MI, Tds, MUM, BM, MP stages of wild-type were sampled for RNA in situ hybridization analysis. The experiment was carried out with reference to the method described [46]. All images were taken using an optical microscope (Zeiss, Oberkochen, Germany).

### Quantitative reverse transcription (qRT)-PCR

Total RNA was extracted, after which the PrimeScript TM RT reagent kit (Takara Bio, Kusatsu, Japan) was used for the cDNA synthesis. qRT-PCR was carried out with SYBR PrimeScript™ RT PCR Kit II (Takara Bio) and sequence-specific primers, with ubiquitin (UBI; serial number: Solyc01g056940) as the reference gene. The relative expression levels of examined genes were computed according to the 2 ^−ΔΔCT^ method with three biological replicates.

### Fruit phenotype analysis

Five plants were randomly selected, and then ten flowers from each plant were randomly selected for fruit set rate counting. The length and width of Br + 7 fruits (7 days after the breaker stage) were measured using a cursor caliper, and single fruit weight was determined using an electronic analytical balance. The number of seeds in the tomato fruits at the Br + 7 stage was also counted.

### Pollen viability assay

Pollen viability was measured using the TTC method [26], and pollen germination media used was according to Yang’s method [26]. Tomato pollen from the wild-type (WT) and *SlHB8* transgenic tomato plants were incubated in PGM at 25°C for 2 h. Images were taken under a Leica microscope (Leica, Wetzlar, Germany). Anthers from the WT and transgenic tomato plants were counterstained with 0.1 mg mL^−1^ DAPI to assess their nuclear status. The DAPI emission signals were 350 nm/460 nm. WT and transgenic lines were crossed as both paternal and maternal plants, after which fruit setting rates were statistically analysed.

### Cytological characterization of anthers

Anthers from different developmental stages (MI, microspore mother cell stage; Tds, tetrad stage; MUM, middle uninucleate microspore stage; BM, binucleate microspore stage; MP, mature pollen stage) were collected during the flowering period and fixed at room temperature for 24–36 h. After paraffin embedding and sectioning, anther cell characteristics were examined using a Leica microscope. A terminal deoxynucleotidyl transferase-mediated biotin-16-dUTP nick-end labeling (TUNEL) assay was carried out using the DeadEnd™ Fluorometric TUNEL System (Promega, Madison, WI, USA), according to its handbook. The images of sections were taken under a Leica TCS SP5 fluorescence confocal scanning microscope. Emission wavelengths of 488 nm/505–545 nm and 561 nm/575–650 nm were used for the TUNEL and propidium iodide signals detection.

### Electron microscopy of pollen phenotypes

For scanning electron microscopy (SEM), mature pollens from *SlHB8* transgenic and wild-type plants were fixed on SEM carriers, coated with gold–palladium. Pollen images were taken under a EVO MA15 scanning electron microscope (Zeiss, Oberkochen, Germany).

Anthers at different developmental stages (MI, Tds, MUM, BM, and MP) were fixed in 4% glutaraldehyde and 2% paraformaldehyde at 4°C overnight. After washing with 0.1 M PBS (four times), the fixed pollen grains were incubated in 1.5% low melting agar, post-fixed in 1% osmium tetroxide, and dehydrated with a graded ethanol series. The samples were then transferred to acetone and embedded in Spurr’s resin (SPI, West Chester, PA, USA). Sections (70 nm thick) were cut using an ultramicrotome (UC7; Leica), collected on copper grids, and stained with uranyl acetate and lead citrate. The stained grids were then photographed with a Talos L120C electron microscope (Thermo Fisher Scientific, Waltham, MA, USA).

### RNA-seq and DNA affinity purification (DAP)-seq analysis

Flower Buds at the tetrad stage were collected and frozen with liquid nitrogen for RNA extraction and transcriptome sequencing experiment which will be carried out by the company Gene Denovo Biotechnology Co., Ltd (Guangzhou, China). Fragments per kilobase of transcript per million mapped reads (FPKM) was used for calculating the expression levels of detected genes. The threshold of log2 (fold change) ≥ 1 and false discovery rate (FDR) ≤ 0.05 were used for defining the differentially expressed genes (DEGs). Transcriptome data analysis and mapping were performed using the online platform OmicShare Tools developed by Gene Denovo (www.omicshare.com/tools). Heatmaps were generated using TBtools as described in the manual [64].

The *SlHB8* gene was cloned into the protein expression halo vector provided by Gene Denovo, the buds at different developmental stages (MI, Tds, MUM, BM, and MP) were graded sampled and mixed at equal ratios, frozen with liquid nitrogen for stock. Protein purification for DAP-seq and extraction and sequencing of genomic DNA were performed by Gene Denovo.

### Yeast one-hybrid assay (Y1H)

The pGADT7-Rec vector with full length CDS of *SlHB8* was treated as a prey vector. pAbAi vector containing multiple SlHB8 binding elements obtained from DAP-seq analysis were regarded as a bait vector. The selection of minimal inhibitory concentration of aureobasidin A was carried out after the transformation of Y1H Gold yeast strains with linearized pAbAi constructs. The binding activity of SlHB8 on the elements was examined by transforming the prey vector to the bait yeast strains which will be cultured on the SD medium lacking Leu (SD/−Leu) with or without aureobasidin A of selected concentration at 30°C for 2–3 days.

### Dual-luciferase transient expression assay

To check the regulatory activity of SlHB8 on the promoters containing SlHB8 binding elements, the pGreenII 62-SK vector containing the *SlHB8* CDS were treated as effector vector, and the pGreenII 0800-LUC vector with target promoters were regarded as reporter vector. These plasmids with differential ratios and composition were injected into *Nicotiana benthamiana* leaves via *Agrobacterium tumefaciens* mediation. The activities of luciferase and *Renilla* were measured using the Dual-Luciferase Assay Kit (Promega) according to its handbook.

### Metabolite analysis

Anthers from flowers at the anthesis stage were collected, frozen in liquid nitrogen for stock. Primary metabolome analysis was performed by MetWare Biotech Ltd. ANOVA (P < 0.01) was used to identify different metabolomes between WT and *SlHB8* transgenic plant.

### Electrophoretic mobility shift assay (EMSA)

The pGEX-4 T-1 vector containing full-length CDS of *SlHB8* was transferred into *Escherichia coli* strain BM Rosetta (DE3) to producing SlHB8-GST fusion protein. The SlHB8 protein purification and EMSA operational approach were according to the methods descried in the publication of Drakakaki [65].

## Acknowledgements

This work was supported by the National Natural Science Foundation of China (31902013 and 31870286); the Natural Science Foundation of Guangdong Province (2018A030310205, 2022A1515012278, 2021A1515010528, and 2017A030313114); and the General Project of Guangzhou City (201804010031). We thank Jilei Huang and Chuanhe Liu from the Instrumental Analysis & Research Center, South China Agricultural University for help with TEM sample processing and image acquisition; Juan Zhou from the Instrumental Analysis & Research Center, South China Agricultural University for help with SEM sample processing and image acquisition; Dr. Hai Zhou from the College of Biology Science of South China Agricultural University for help with TUNEL experiment.

## Author contributions

C.W., Y.Y., Z.X., C.Y., Z.P., H.G., and D.S. performed the research; Y.H., R.C., and Z.L. design of the research. Y.H., G.H., D.C., and C.W. analysed the data, Y.H. and C.W. wrote the manuscript. All authors assisted with manuscript revision. All authors read and approved the final version of the manuscript.

## Data availability

All data generated or analysed during this study are included in this published article and its supplementary information files.

## Conflict of interest

The authors declare no conflicts of interest.

## Supplementary data

Supplementary data is available at *Horticulture Research * online.

## Supplementary Material

Web_Material_uhac185
