# Supplementary material for: The SlHB8 acts as a negative regulator in tapetum development and pollen wall formation in Tomato
Source: Hortic Res. 2022 Aug 25;9:uhac185. doi: 10.1093/hr/uhac185 (PMC9627519; doi:10.1093/hr/uhac185)
Supplement: Web_Material_uhac185 [file web_material_uhac185.zip › Supplementary file revised with yellow highlights.pdf]

Supplementary Information

Article title: The SlHB8 acts as a negative regulator in tapetum development and pollen wall formation

Authors: Caiyu Wu, Yang Yang, Deding Su, Canye Yu, Zhiqiang Xian, Zanlin Pan, Hongling Guan, Guojian Hu, Da Chen, Zhengguo Li, Riyuan Chen, Yanwei Hao

The following Supporting Information is available for this article:

Figure S1–S9

Table S1–S7

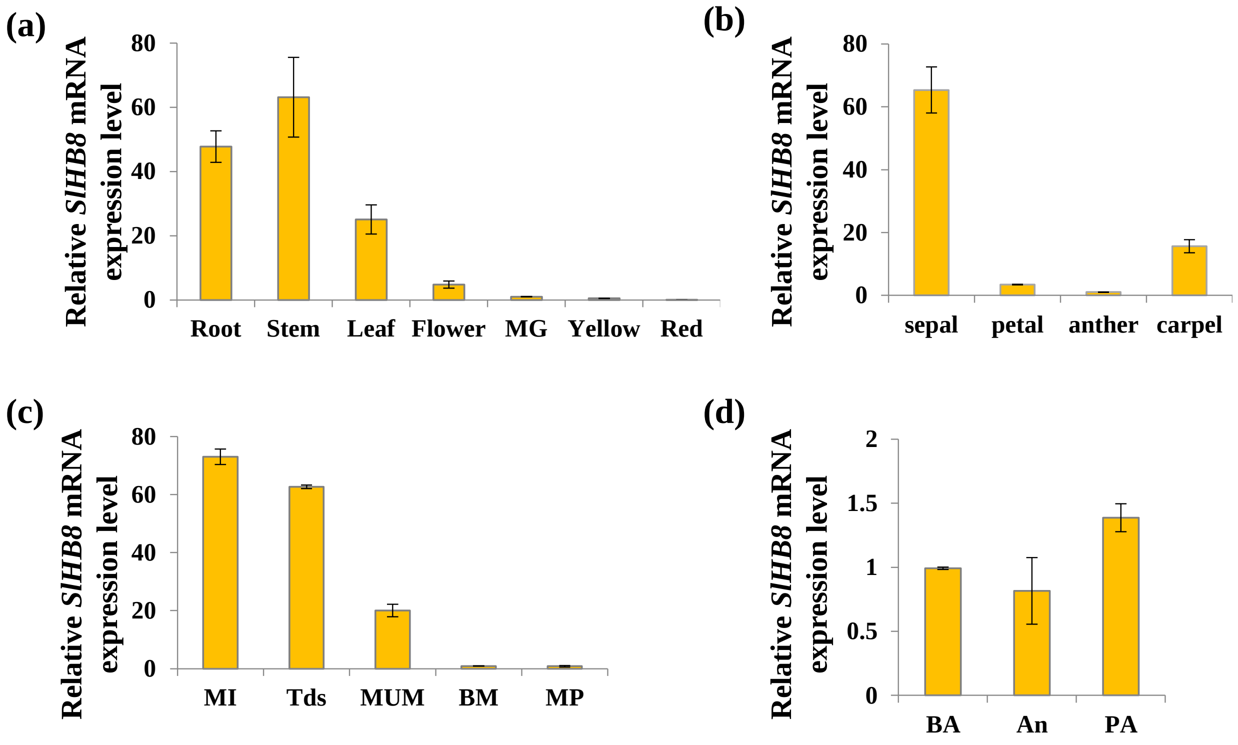

Figure S1. Expression pattern of *SlHB8* during tomato development.

(a) Expression pattern of *SlHB8* in various micro-Tom tomato organs. The expression level in red rip fruit was referred to as one. **(b)** Expression levels of *SlHB8* in the wild-type flower organs at the anthesis stage. The expression level in anther was referred to as one. **(c)**. Expression levels of *SlHB8* in tomato anthers at different development stages. The expression level in MP anther was referred to as one. MI, anther at microspore mother cell stage; Tds, anther at tetrad stage; MUM, anther at middle uninucleate microspore stage; BM, anther at binucleate microspore stage; MP, anther at mature pollen stage. **(d)** Expression levels of *SlHB8* in the ovaries of wild-type tomato during fruit setting. The expression level in ovary before anthsis was referred to as one. *Ubi* was used as a reference gene. The yellow bar chart represents the expression level of *SlHB8*. BA: ovaries at two days before anthsis stage. An: ovaries at anthesis stage. PA: ovaries at 4 days post anthesis stage.

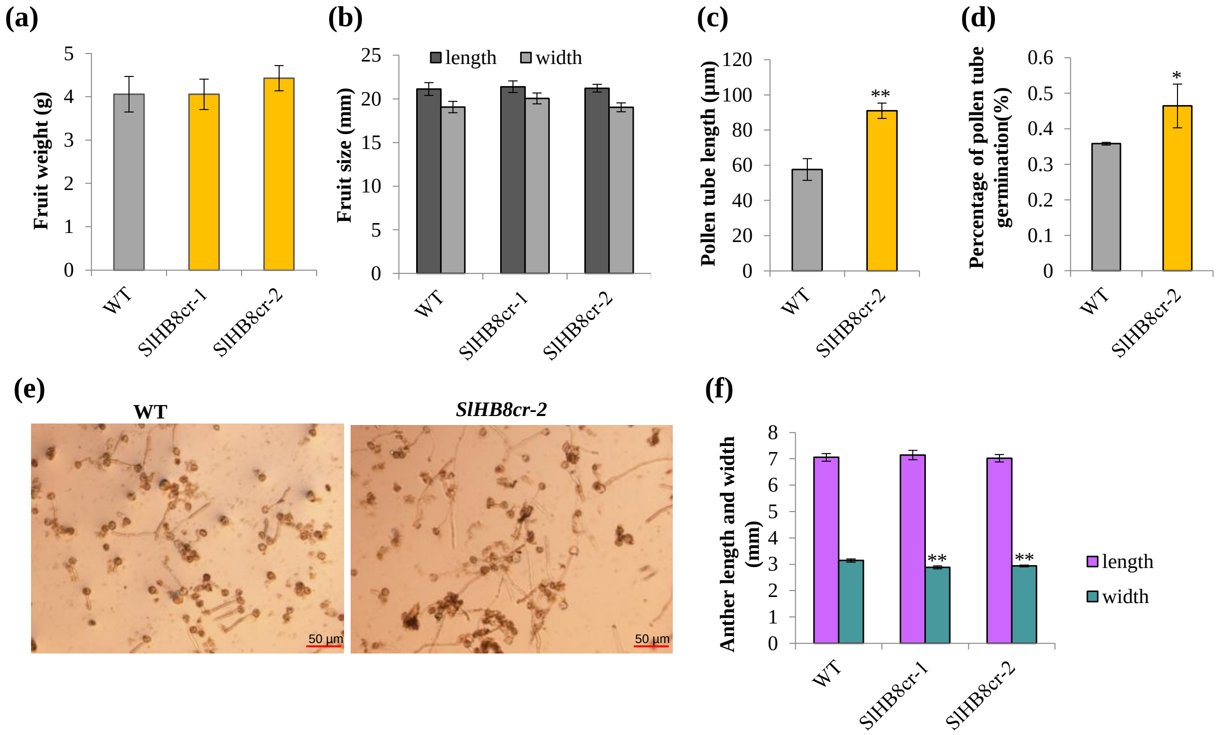

Figure S2. Fruits and flower phenotypes of *SlHB8* knockout plants.

Fruit weight (**a**)**,** fruit size **(b)**, pollen tube length (**c**), pollen tube germination rate (**d**,**e**) and anther size (**f**) of wild-type and *SlHB8* knockout plants. Error bar = SE; **P* < 0.5, ***P* < 0.01 (Student's *t*-test; compared with the WT). Scale Bar = 50 µm.

Click here to enter text.

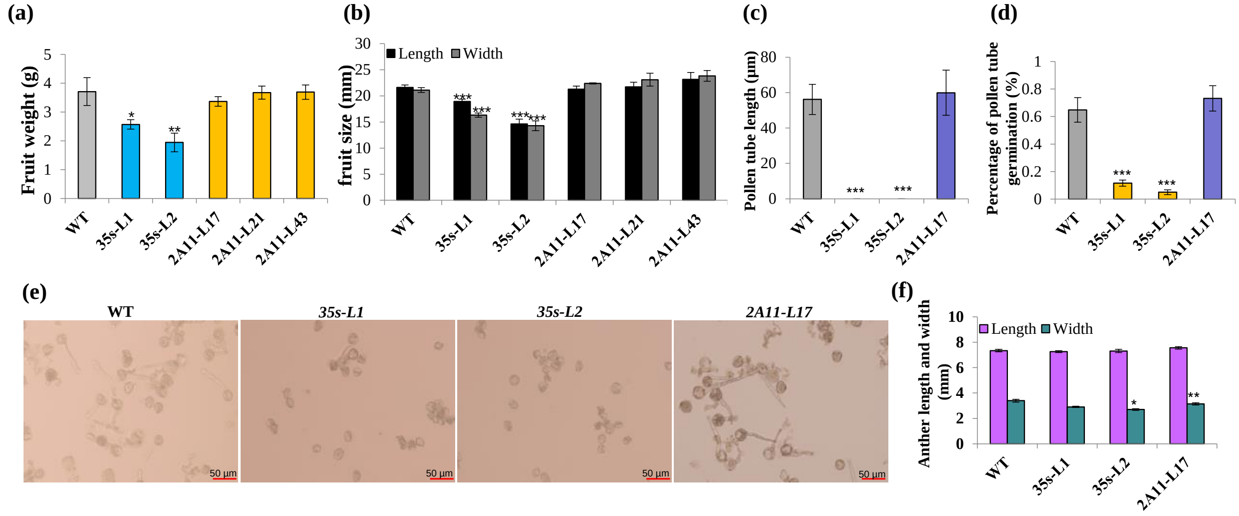

Figure S3. Fruits and flower phenotypes of *SlHB8* overexpression plants.

Fruit weight (**a**), fruit size (**b**), pollen tube length (**c**), pollen tube germination rate (**d**,**e**) and anther size (**f**) of wild-type and *SlHB8* gene overexpression plants. Error bar = SE; statistical analysis was performed using the Students' *t*-test, **P* < 0.5, ***P* < 0.01, ****P*<0.001(Student's *t*-test; compared with the WT). *35s-L1*: promoter 35s drive *SlHB8* overexpression line 1. *2A11-L17*: promoter 2A11 drive *SlHB8* overexpression line 17. Scale Bar = 50 μm.

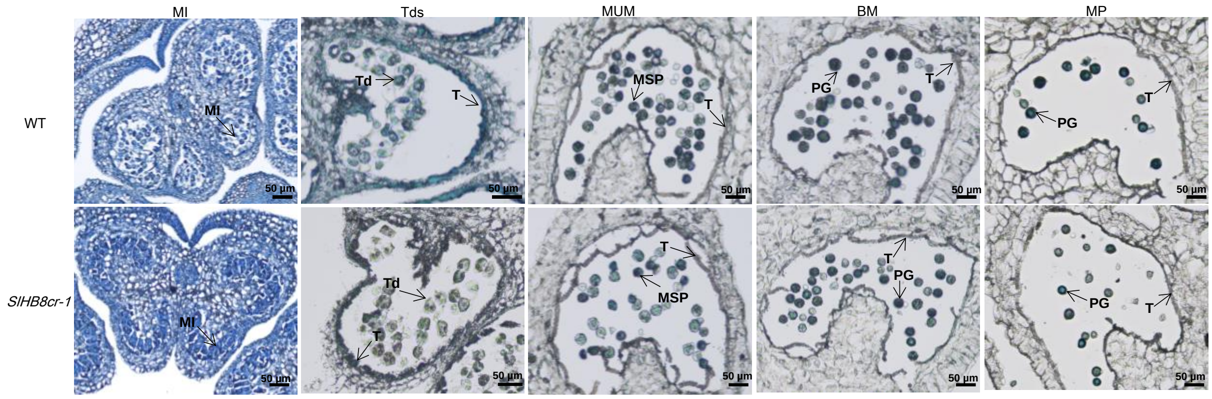

Figure S4. Histocytological observation of pollen development in the wild-type and *SlHB8* gene knockout plant.

Semi-thin section comparison of anther and pollen development between the wild-type and *SlHB8* gene knockout plant. MI, microspore mother cell stage; Tds, tetrad stage; MUM, Middle uninucleate microspore stage; BM, binucleate microspore stage; MP, mature pollen stage; T: tapetum; Td: tetrad; MSP: microspore; PG: pollen grain; APG: abnormal pollen grain. The scale bar indicated 50 μm.

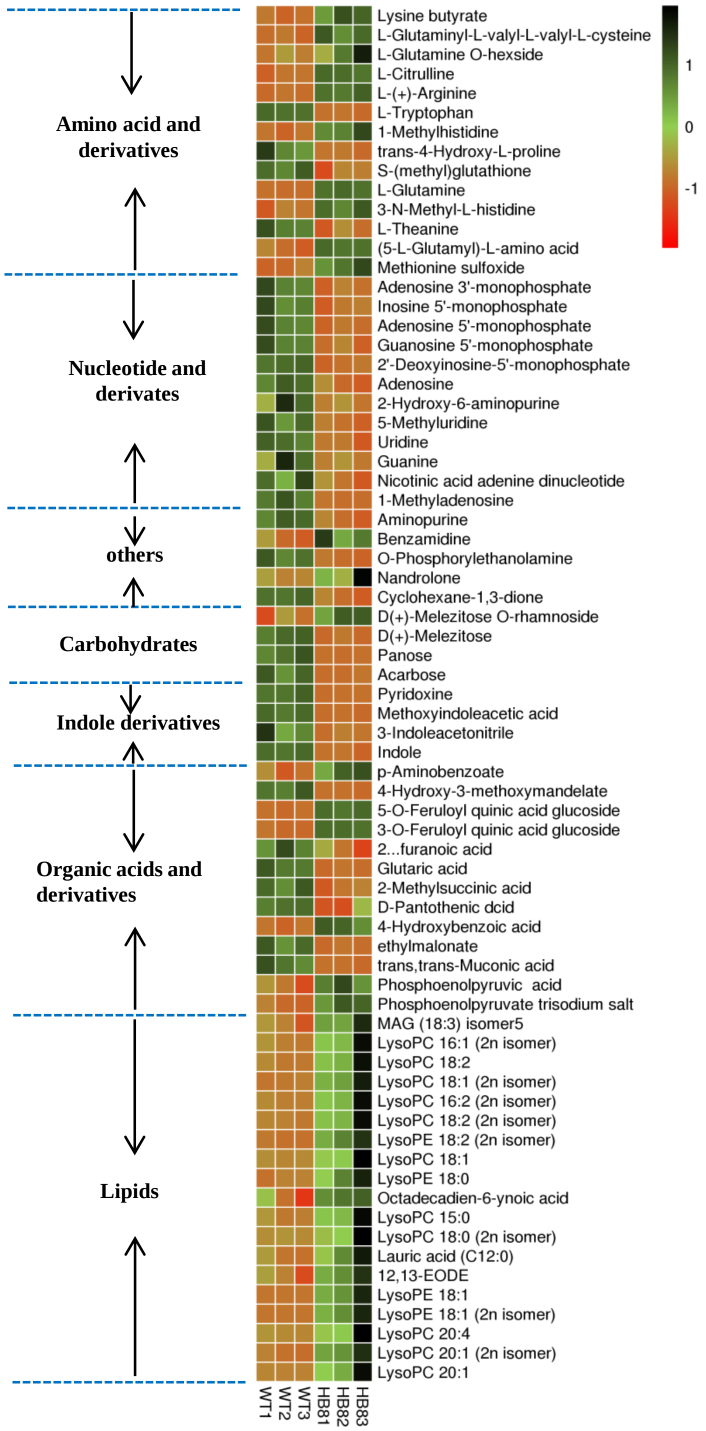

Figure S5. Levels of the primary metabolites amino acids, lipids, nucleotides, organic acids, carbohydrates, and indole in the mature pollen stage anthers of wild-type and *SlHB8-ox* transgenic plants.

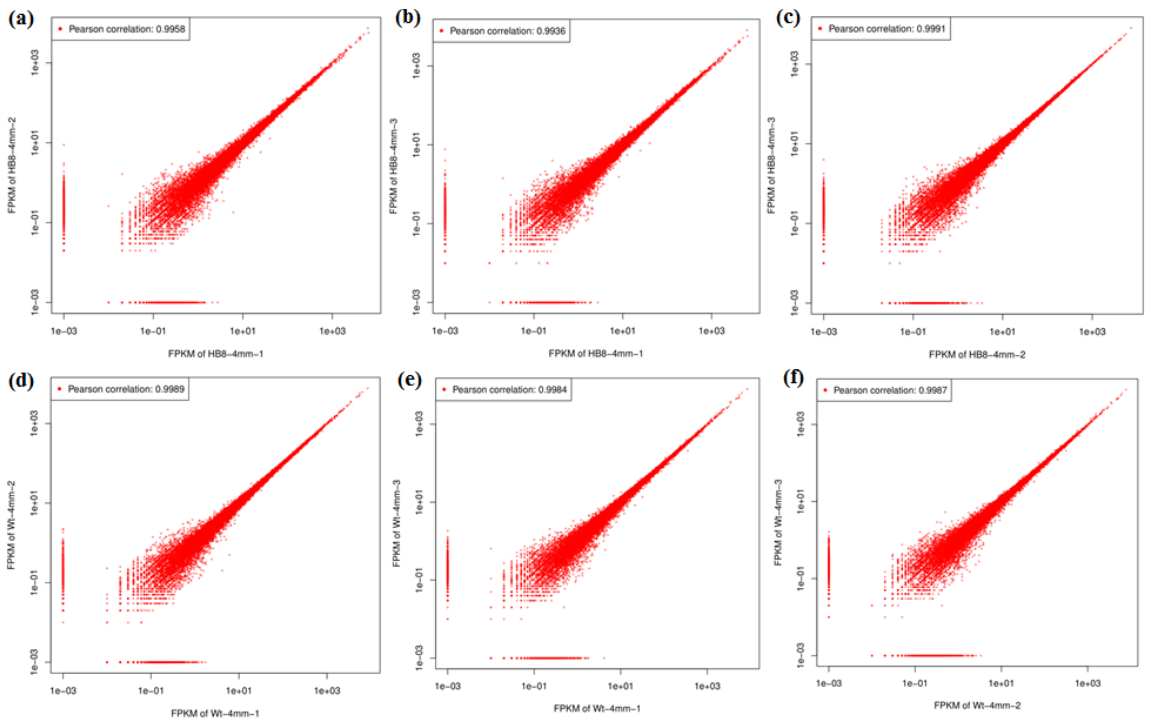

Figure S6. Correlation between gene expression levels in the wild-type and *p35s::SlHB8Ris* anthers at the tetrad stage.

Results of three biological replicates were analyzed. Pearson's correlation coefficient of HB8-4mm-1 vs. HB8-4mm-2 **(a)**; HB8-4mm-1 vs. HB8-4mm-3 **(b)**; HB8-4mm-3 vs. HB8-4mm-2 **(c)**; WT-4mm-1 vs. WT-4mm-2 **(d)**; WT-4mm-1 vs. WT-4mm-3 **(e)**; and WT-4mm-3 vs. WT-4mm-2 **(f)**. R^2 > 0.8 was the significance cutoff.

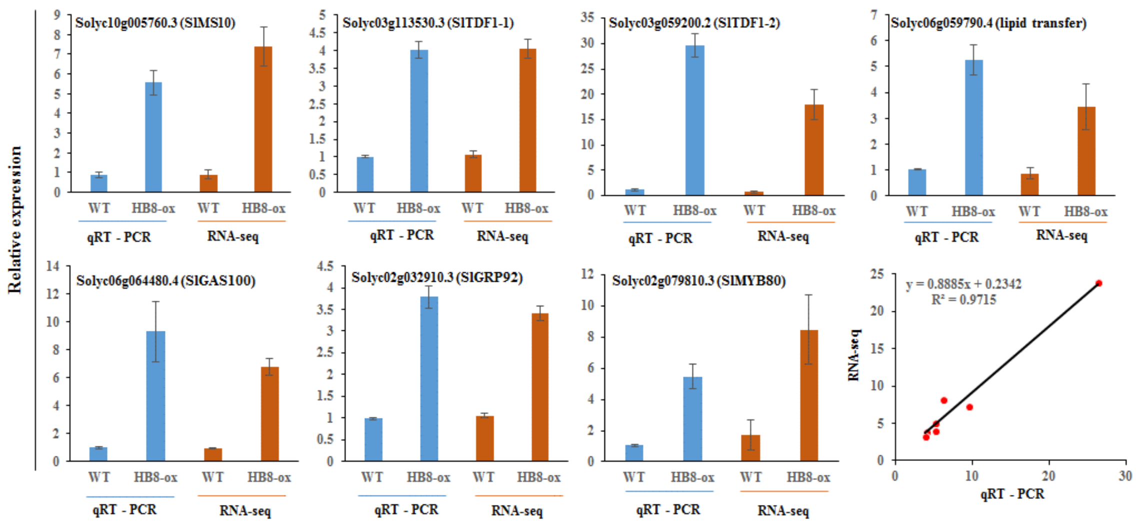

Figure S7. Validation of the RNA-seq expression data using qRT-PCR.

(a) Relative expression levels of eight genes determined by qRT-PCR and the FPKM obtained by RNA-seq. Results for each gene are based on three biological and three technical replicates. *Ubi* was used as the reference gene. Error bars indicate SE. **(b)** Pearson's correlation coefficient of RNA-seq and qRT-PCR data was insignificant; R^2 > 0.8 was the significance threshold. The values are the row Z-score of RPKM and qRT-PCR.

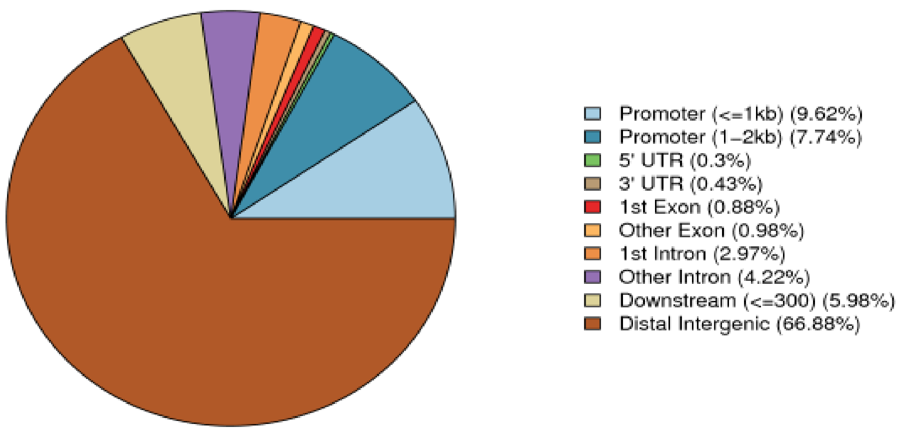

Figure S8. Peak distribution of the SlHB8-binding element in the tomato genome.

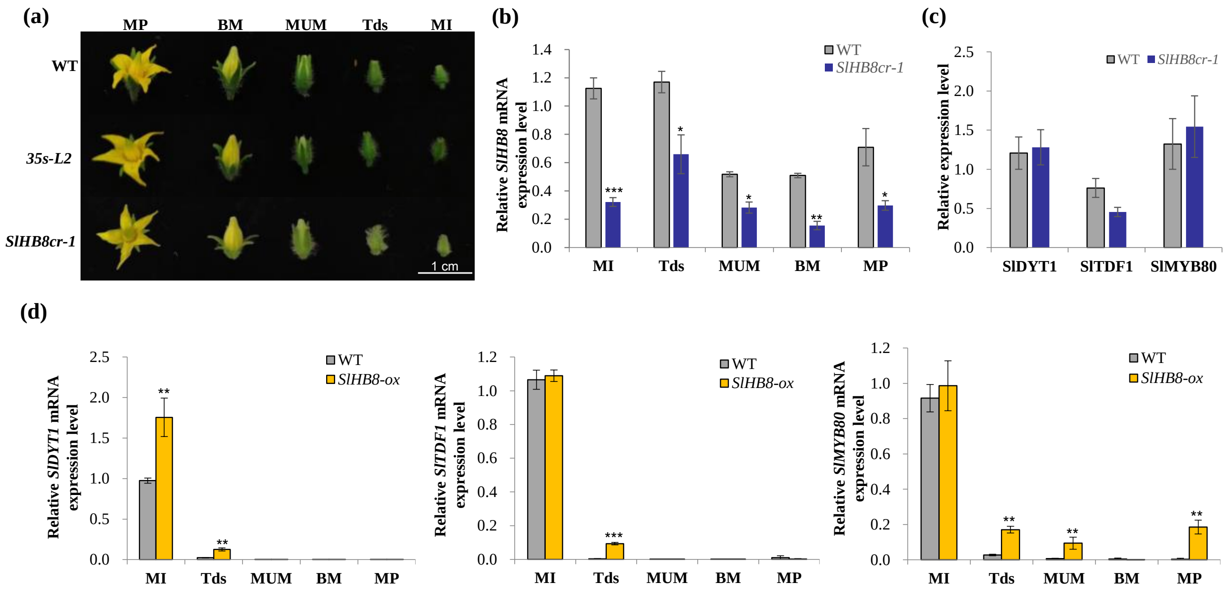

Figure S9. Expression pattern of *SlHB8* and tapetum PCD related genes during pollen development.

(a) Flower bud photos of wild-type, *SlHB8* overexpression and *SlHB8* gene knockout plants. Scale bar = 1 cm. **(b)** Expression pattern of *SlHB8* in wild-type and *SlHB8* gene knockout plant during pollen development. (**c**) Expression level of tapetum PCD regulators in anther tissues at tetrad stage in wild-type and *SlHB8* gene knockout plant during pollen development. *Ubi* was used as a reference gene. Error bar = SE. **(d)** Expression levels of tapetum PCD related genes in the wild-type and *SlHB8* overexpression plants during pollen development. The expression level in MI was referred to as one. *Ubi* was used as a reference gene. MI, anthers at microspore mother cell stage; Tds, anthers at tetrad stage; MUM, anthers at middle uninucleate microspore stage; BM, anthers at binucleate microspore stage; MP, anthers at mature pollen stage. *SlDYT1*, *SlTDF1*, *SlMYB80* were homologues genes of *DYT1*, *TDF1* and *MYB80* in Arabidopsis. Error bar = SE; statistical analysis was performed using the Students' *t*-test, **P* < 0.5, ***P* < 0.01, ****P*<0.001(Student's *t*-test; compared with the WT).

Table S1. Amino acid, lipid, nucleotide, organic acid, carbohydrate, and indole content in the anthers of wild-type and *SlHB8-ox* lines

Index	Compounds	Class	WT	SlHB8-OX	VIP	Fold_Change
pmb0962	Lysine butyrate	Amino acid and derivatives	8.14E+05	2.09E+06	1.29E+00	2.57
pmb1283	L-Glutaminyl-L-valyl-L-valyl-L-cysteine	Amino acid and derivatives	9.14E+04	2.30E+05	1.30E+00	2.52
pmb2855	L-Glutamine O-hexside	Amino acid and derivatives	1.80E+06	4.51E+06	1.09E+00	2.50
pme0007	L-Citrulline	Amino acid and derivatives	1.46E+06	3.11E+06	1.30E+00	2.12
pme0043	L-(+)-Arginine	Amino acid and derivatives	7.52E+06	3.52E+07	1.31E+00	4.68
pme0050	L-Tryptophan	Amino acid and derivatives	1.09E+07	3.14E+06	1.31E+00	0.29
pme0181	1-Methylhistidine	Amino acid and derivatives	4.03E+05	1.20E+06	1.29E+00	2.97
pme0247	trans-4-Hydroxy-L-proline	Amino acid and derivatives	1.97E+05	6.62E+04	1.28E+00	0.34
pme1242	S-(methyl)glutathione	Amino acid and derivatives	9.11E+05	4.35E+05	1.25E+00	0.48
pme1408	L-Glutamine	Amino acid and derivatives	1.38E+06	2.85E+06	1.31E+00	2.06
pme2046	3-N-Methyl-L-histidine	Amino acid and derivatives	2.70E+05	6.86E+05	1.28E+00	2.54
pme2142	L-Theanine	Amino acid and derivatives	8.83E+04	3.60E+04	1.26E+00	0.41
pme2569	(5-L-Glutamyl)-L-amino acid	Amino acid and derivatives	9.24E+05	1.90E+06	1.29E+00	2.05
pme2617	Methionine sulfoxide	Amino acid and derivatives	6.94E+05	1.47E+06	1.28E+00	2.11
pmb0514	Adenosine 3'-monophosphate	Nucleotide and derivates	8.81E+05	3.15E+05	1.28E+00	0.36
pmb0532	Inosine 5'-monophosphate	Nucleotide and derivates	3.10E+06	1.23E+06	1.28E+00	0.40
pmb0981	Adenosine 5'-monophosphate	Nucleotide and derivates	9.18E+05	3.16E+05	1.30E+00	0.34
pmb0998	Guanosine 5'-monophosphate	Nucleotide and derivates	2.00E+06	7.22E+05	1.28E+00	0.36
pmc0066	2'-Deoxyinosine-5'-monophosphate	Nucleotide and derivates	4.84E+05	2.21E+05	1.30E+00	0.46
pmd0023	Adenosine	Nucleotide and derivates	5.21E+06	2.38E+06	1.27E+00	0.46
pme0183	2-Hydroxy-6-aminopurine	Nucleotide and derivates	6.91E+06	2.56E+06	1.11E+00	0.37
pme0263	5-Methyluridine	Nucleotide and derivates	3.52E+05	1.43E+05	1.29E+00	0.41
pme1063	Uridine	Nucleotide and derivates	6.90E+06	2.23E+06	1.28E+00	0.32
pme1109	Guanine	Nucleotide and derivates	7.19E+06	2.74E+06	1.09E+00	0.38
pme1363	Nicotinic acid adenine dinucleotide	Nucleotide and derivates	5.79E+04	1.63E+04	1.20E+00	0.28
pme1378	1-Methyladenosine	Nucleotide and derivates	4.23E+05	1.49E+05	1.30E+00	0.35
pmb0374	Aminopurine	Others	1.98E+06	8.73E+05	1.28E+00	0.44
pme1011	Benzamidine	Others	5.77E+04	1.19E+05	1.22E+00	2.07
pme2830	O-Phosphorylethanolamine	Others	1.71E+07	7.07E+06	1.30E+00	0.41
pmf0504	Nandrolone	Others	1.69E+04	4.58E+04	1.04E+00	2.72

pmf0537	Cyclohexane-1,3-dione	Others	7.36E+05	3.66E+05	1.29E+00	0.50
pmb2653	D(+)-Melezitose O-rhamnoside	Carbohydrates	2.58E+04	5.22E+04	1.22E+00	2.02
pme0500	D(+)-Melezitose	Carbohydrates	1.40E+05	3.38E+04	1.30E+00	0.24
pmf0485	Panose	Carbohydrates	1.59E+05	6.14E+04	1.30E+00	0.38
pmf0576	Acarbose	Carbohydrates	1.00E+05	2.49E+04	1.30E+00	0.25
pme1383	Pyridoxine	Vitamins and derivatives	6.18E+06	9.71E+05	1.31E+00	0.16
pmb0818	Methoxyindoleacetic acid	Indole derivatives	9.31E+06	2.49E+06	1.31E+00	0.27
pmb0819	3-Indoleacetonitrile	Indole derivatives	1.90E+06	6.28E+05	1.28E+00	0.33
pmb1096	Indole	Indole derivatives	9.25E+06	2.42E+06	1.30E+00	0.26
pmb0247	p-Aminobenzoate	Organic acids and derivatives	1.12E+06	2.50E+06	1.24E+00	2.24
pmb2497	4-Hydroxy-3-methoxymandelate	Organic acids and derivatives	2.87E+05	1.18E+05	1.31E+00	0.41
pmb2554	5-O-Feruloyl quinic acid glucoside	Organic acids and derivatives	2.38E+05	7.16E+05	1.31E+00	3.01
pmb2833	3-O-Feruloyl quinic acid glucoside	Organic acids and derivatives	1.99E+05	5.71E+05	1.31E+00	2.87
pme0238	2–furanoic acid	Organic acids and derivatives	6.45E+05	2.98E+05	1.16E+00	0.46
pme0243	Glutaric acid	Organic acids and derivatives	3.89E+06	1.20E+06	1.31E+00	0.31
pme0267	2-Methylsuccinic acid	Organic acids and derivatives	1.54E+05	5.69E+04	1.27E+00	0.37
pme1683	D-Pantothenic dcid	Organic acids and derivatives	4.08E+06	1.82E+06	1.17E+00	0.45
pme2903	4-Hydroxybenzoic acid	Organic acids and derivatives	3.38E+05	8.45E+05	1.30E+00	2.50
pme3034	ethylmalonate	Organic acids and derivatives	1.07E+05	3.58E+04	1.30E+00	0.33
pme3207	trans,trans-Muconic acid	Organic acids and derivatives	1.03E+06	3.07E+05	1.30E+00	0.30
pmf0196	Phosphoenolpyruvic acid	Organic acids and derivatives	3.65E+05	9.33E+05	1.21E+00	2.55
pmf0218	Phosphoenolpyruvate trisodium salt	Organic acids and derivatives	1.24E+05	4.55E+05	1.28E+00	3.67
pmb0160	MAG (18:3) isomer5	Lipids	1.74E+07	3.58E+07	1.18E+00	2.05
pmb0848	LysoPC 16:1 (2n isomer)	Lipids	5.56E+06	1.25E+07	1.15E+00	2.25
pmb0852	LysoPC 18:2	Lipids	9.58E+05	2.99E+06	1.20E+00	3.12
pmb0859	LysoPC 18:1 (2n isomer)	Lipids	2.68E+06	2.25E+07	1.28E+00	8.40
pmb0863	LysoPC 16:2 (2n isomer)	Lipids	5.73E+06	2.09E+07	1.21E+00	3.64
pmb0873	LysoPC 18:2 (2n isomer)	Lipids	8.42E+05	2.65E+06	1.20E+00	3.14
pmb0874	LysoPE 18:2 (2n isomer)	Lipids	4.45E+05	2.78E+06	1.28E+00	6.26
pmb0882	LysoPC 18:1	Lipids	3.78E+05	1.12E+06	1.11E+00	2.96
pmb0883	LysoPE 18:0	Lipids	1.80E+05	4.82E+05	1.21E+00	2.68
pmb1574	Octadecadien-6-ynoic acid	Lipids	2.46E+05	4.96E+05	1.10E+00	2.02
pmb2319	LysoPC 15:0	Lipids	6.74E+05	1.73E+06	1.15E+00	2.57
pmb2388	LysoPC 18:0 (2n isomer)	Lipids	1.05E+05	3.39E+05	1.09E+00	3.23
pmb2640	Lauric acid (C12:0)	Lipids	5.12E+03	1.15E+04	1.14E+00	2.24
pmb2799	12,13-EODE	Lipids	9.83E+05	2.27E+06	1.14E+00	2.31
pmb3121	LysoPE 18:1	Lipids	4.99E+04	7.42E+05	1.30E+00	14.88
pmb3132	LysoPE 18:1 (2n isomer)	Lipids	5.65E+04	8.73E+05	1.30E+00	15.45

pmc0960	LysoPC 20:4	Lipids	3.56E+06	1.02E+07	1.09E+00	2.86
pmd0144	LysoPC 20:1 (2n isomer)	Lipids	9.14E+04	3.67E+05	1.27E+00	4.01
pmd0145	LysoPC 20:1	Lipids	3.19E+05	1.78E+06	1.23E+00	5.57

Table S2. Summary statistics of RNA-seq data of the six libraries mapped to the tomato reference genome (*Solanum lycopersicum* ITAG4.0).

Stage	WT-4mm			SlHB8-ox-4mm		
Library	WT-4mm-1	WT-4mm-2	WT-4mm-3	SlHB8-4mm-1	SlHB8-4mm-2	SlHB8-4mm-3
raw reads	58280618	44228032	36543606	42664096	36622150	43549122
High quality clean reads (%)	58217074 (99.89%)	44180000 (99.89%)	36500558 (99.88%)	42622688 (99.90%)	36583094 (99.89%)	43504790 (99.90%)
Removed rRNA reads (%)	55163516 (94.75%)	42329498 (95.81%)	35520704 (97.32%)	41446288 (97.24%)	54120960 (95.21%)	42689616 (98.13%)
Mapped reads(%)	53728984 (97.40%)	41185529 (97.30%)	34583039 (97.36%)	40353357 (97.36%)	34937320 (97.17%)	41635754 (97.53%)
Unique mapped reads (%)	51853823 (94.00%)	39806823 (94.04%)	33465616 (94.21%)	39106102 (94.35%)	33861004 (94.18%)	40343010 (94.50%)
Multiple mapped reads	1875161 (3.40%)	1378706 (3.26%)	1117423 (3.15%)	1247255 (3.01%)	1076316 (2.99%)	1292744 (3.03%)
All genes	24399 (70.09%)	24030 (69.03%)	23723 (68.15%)	23974 (68.87%)	23930 (68.74%)	24109 (69.26%)
Known genes	23733 (69.65%)	23358 (68.55%)	23064 (67.69%)	23297 (68.37%)	23262 (68.27%)	23435 (68.77%)
Novel transcripts	666	672	659	677	668	674

Table S3. Differentially expressed genes between the wild-type and *SlHB8* overexpression lines.

Table S4. Gene ontology and KEGG functional analysis of 545 up-regulated differentially expressed genes.

Table S5. SlHB8 DAP seq data file. Click here to enter text.

Table S6. RNA-seq and DAP-seq overlapping differentially expressed genes.

Table S7. Genes in wild type and *SlHB8* overexpression line.
